# Robot-assisted rehabilitation for post-stroke balance and postural control: an umbrella review and evidence map of systematic reviews

**DOI:** 10.3389/fneur.2026.1911635

**Published:** 2026-09-08

**Authors:** Ying Liu, Qian Hu, Shao Yin, Yue Zhong, Qiang Ji, Fengya Zhu

**Affiliations:** 1 The Second People’s Hospital of Neijiang, Neijiang, China; 2 Neijiang Hospital of Traditional Chinese Medicine, Neijiang, China; 3 Zigong First People’s Hospital, Zigong, China

**Keywords:** balance, evidence map, postural control, robot-assisted rehabilitation, stroke, umbrella review

## Abstract

**Objective:**

Balance and postural-control impairments are common after stroke and can restrict mobility and independence, but the effectiveness of robot-assisted rehabilitation for these outcomes remains uncertain. This umbrella review aimed to summarize and critically appraise evidence from systematic reviews on robot-assisted rehabilitation for balance function and postural control after stroke.

**Methods:**

This umbrella review was prospectively registered in PROSPERO (CRD420261426090). PubMed, Embase, the Cochrane Library, and Web of Science were searched from inception to March 30, 2026. Eligible publications were systematic reviews evaluating robot-assisted rehabilitation in adults after stroke and reporting at least one balance- or postural-control-related outcome. Methodological quality, reporting completeness, risk of bias, certainty of evidence, and primary-study overlap were assessed using AMSTAR-2, PRISMA 2020, ROBIS, GRADE, and corrected covered area (CCA), respectively.

**Results:**

Twelve systematic reviews were included. The included reviews evaluated robot-assisted gait training, Lokomat, overground robot-assisted gait training, end-effector robots, exoskeleton robots, lower-limb rehabilitation robots, and mixed robotic devices. The Berg Balance Scale (BBS) was the most frequently reported outcome. Several reviews suggested favorable effects of robot-assisted rehabilitation on BBS, whereas findings for Timed Up and Go, Fugl-Meyer balance, Rivermead Mobility Index, and instrumented postural-control outcomes were less consistent. AMSTAR-2 rated 10 reviews as low quality and two as critically low quality. ROBIS indicated one review with high overall risk of bias and the remaining reviews with unclear risk of bias. The certainty of evidence for major outcomes was generally low or very low. Primary-study overlap was high for overall balance-related evidence (CCA = 12.06%), BBS evidence (CCA = 11.61%), and TUG/TUGT evidence (CCA = 11.76%).

**Conclusion:**

Robot-assisted rehabilitation may improve selected balance-related outcomes after stroke, particularly BBS. However, current evidence is limited by low methodological quality, unclear or high risk of bias, low or very low certainty, heterogeneity, and primary-study overlap. Robot-assisted rehabilitation should be considered a potential adjunct to comprehensive post-stroke rehabilitation, and higher-quality evidence is needed to clarify its effects on balance and postural control.

**Systematic review registration:**

https://www.crd.york.ac.uk/PROSPERO/view/CRD420261426090, PROSPERO (CRD420261426090).

## 1 Introduction

Balance impairment and postural-control dysfunction are common and clinically important sequelae after stroke. Previous studies have suggested that approximately 40–50% of stroke survivors have impaired balance, and some estimates indicate that balance problems may occur in an even larger proportion of patients after stroke (1–4). These impairments are closely associated with reduced walking ability, impaired transfers, activities-of-daily-living (ADL) dependence, fear of falling, fall risk, and reduced health-related quality of life (2, 5, 6). Even among patients with mild stroke or relatively preserved motor function, balance limitations may persist and contribute to reduced mobility and higher fall risk compared with healthy controls (7). Therefore, balance rehabilitation is a core component of post-stroke functional recovery and should not be regarded merely as a secondary extension of gait training.

Post-stroke balance dysfunction is multifactorial and involves related but distinct constructs. Balance refers to the ability to maintain or regain stability during static or dynamic tasks, whereas postural control encompasses the sensory and motor processes that regulate body orientation and stability. Functional mobility additionally reflects the ability to perform transfers, walking, turning, and other goal-directed activities. After stroke, these functions may be disrupted by asymmetric weight bearing, impaired lower-limb support, abnormal postural sway, altered gait symmetry, impaired trunk control, reduced anticipatory and reactive postural adjustments, and impaired integration of proprioceptive, vestibular, and visual inputs (8–13). Motor planning, attention, and cognitive–motor coordination further influence the translation of postural control into safe functional mobility. Clinical evidence suggests that targeted balance training, trunk-stability training, and integrated balance–gait interventions can improve balance, mobility, and activities of daily living after stroke (14–18). Proprioceptive training combined with dual-task exercises may also improve walking ability and selected gait parameters in people with chronic stroke (19). However, conventional gait training alone may be insufficient to address these interdependent deficits, and rehabilitation may need to challenge postural control, trunk stability, weight transfer, sensory integration, and dynamic balance (20–23).

Robot-assisted rehabilitation has been increasingly used in post-stroke lower-limb and gait rehabilitation. Devices such as exoskeletons, end-effector systems, Lokomat, overground robotic systems, and other lower-limb rehabilitation robots can deliver high-intensity, repetitive, task-oriented, and quantifiable practice (24–26). Depending on device design and training mode, robotic systems can combine guided stepping and weight transfer with augmented sensory feedback. These features may support motor learning and sensorimotor integration rather than provide gait assistance alone. Existing reviews and clinical studies suggest potential benefits for walking ability, gait speed, lower-limb motor function, balance, and activities of daily living, although effects on gait coordination and some mobility outcomes remain inconsistent (26–29). However, the available reviews differ in device type, training protocol, comparator, stroke population, outcome construct, and analytic methods, while the extent of primary-study overlap remains unclear. An umbrella review was therefore needed to integrate these heterogeneous review-level findings, evaluate whether apparently concordant conclusions were based on independent bodies of evidence, and compare their methodological credibility and certainty. Accordingly, this umbrella review aimed to synthesize and critically appraise systematic reviews and meta-analyses of robot-assisted rehabilitation for post-stroke balance and postural control. It also mapped the evidence across robotic approaches and outcome domains and evaluated methodological quality, reporting completeness, risk of bias, certainty of evidence, and primary-study overlap.

## 2 Methods

### 2.1 Study design and reporting guidance

This study was designed as an umbrella review of systematic reviews and meta-analyses with an evidence-mapping component (30, 31). The protocol was prospectively registered in PROSPERO (registration number: CRD420261426090). The review aimed to summarize and critically appraise the existing review-level evidence on robot-assisted rehabilitation for balance function and postural control after stroke. The conduct and reporting of this umbrella review were guided by methodological recommendations for overviews of reviews and the Preferred Reporting Items for Systematic Reviews and Meta-Analyses 2020 (PRISMA 2020) statement (32). For each included review, methodological quality was assessed using AMSTAR-2 (33), reporting completeness was summarized according to PRISMA 2020 (32), risk of bias was evaluated using ROBIS (34), certainty of evidence for major outcomes was judged using the GRADE approach (35, 36), and primary-study overlap was quantified using the corrected covered area (CCA) (37, 38).

### 2.2 Search strategy

A comprehensive literature search was conducted in PubMed, Embase, the Cochrane Library, and Web of Science from database inception to March 30, 2026. The search strategy combined controlled vocabulary and free-text terms related to stroke, robot-assisted rehabilitation, robotic gait training, lower-limb robotic devices, balance, postural control, and systematic reviews or meta-analyses. Search terms included stroke, cerebrovascular accident, post-stroke, robot-assisted rehabilitation, robot-assisted therapy, robot-assisted gait training, robotic gait training, electromechanical-assisted gait training, exoskeleton, end-effector, Lokomat, balance, postural control, postural balance, Berg Balance Scale, Timed Up and Go, systematic review, and meta-analysis. The reference lists of relevant reviews were also checked to identify additional potentially eligible publications. The detailed database-specific search strategies are provided in Supplementary material.

### 2.3 Eligibility criteria

Eligibility criteria were prespecified according to the Population, Intervention, Comparator, Outcomes, and Study design (PICOS) framework. The population (P) comprised adults with ischemic or hemorrhagic stroke; cerebrovascular accident and post-stroke hemiplegia were accepted as equivalent population descriptors. The intervention (I) was robot-assisted lower-limb, gait, balance, or postural-control rehabilitation, delivered alone or in addition to conventional rehabilitation. Eligible comparators (C) included conventional physiotherapy or rehabilitation, usual care, non-robotic gait training, treadmill training, another non-robotic intervention, or no robot-assisted intervention. Eligible outcomes (O) were separately reported measures of balance, postural control, or balance-related functional mobility. Eligible study designs (S) were systematic reviews with or without pairwise meta-analysis and network meta-analyses. Reviews of mixed neurological populations were eligible only when stroke-specific findings could be extracted or stroke patients constituted the target population of the relevant robotic rehabilitation evidence.

Robot-assisted rehabilitation encompassed lower-limb exoskeletons, wearable or overground exoskeletons, treadmill-based robotic gait systems, end-effector robots, lower-limb rehabilitation robots, and other electromechanical devices used for post-stroke lower-limb, gait, balance, or postural-control training. Lokomat, Gait Trainer, and G-EO were treated as examples of eligible robotic systems rather than as separate eligibility categories.

Eligible outcome measures included, but were not limited to, the Berg Balance Scale (BBS), Timed Up and Go test (TUG/TUGT), Fugl-Meyer balance scale (FMB), Postural Assessment Scale for Stroke (PASS), Functional Reach Test (FRT), Standing Functional Reach Test (SFRT), Tinetti Performance-Oriented Mobility Assessment (POMA), Trunk Impairment Scale (TIS), Activities-specific Balance Confidence scale (ABC), postural sway, center of pressure (COP), postural stability, and instrumented postural-control measures. Balance, postural control, and functional mobility were treated as related but distinct constructs; functional-mobility measures such as TUG/TUGT were therefore retained as a separate outcome domain rather than interpreted as direct equivalents of balance or postural-control measures. Reviews primarily focusing on lower-limb motor function, walking ability, or robotic gait training were eligible only when at least one relevant balance-, postural-control-, or balance-related functional-mobility outcome was reported separately.

We excluded primary clinical trials, observational studies, case reports, case series, narrative reviews, scoping reviews, guidelines, protocols, conference abstracts, letters, editorials, and animal or mechanistic studies. Reviews were also excluded if they did not involve stroke patients, did not evaluate robot-assisted rehabilitation, did not report any balance- or postural-control-related outcome, or did not allow the effect of robot-assisted rehabilitation to be separated from a complex non-robotic intervention package. Duplicate or superseded reviews were excluded from the main appraisal when a more recent or more comprehensive review addressed the same topic with overlapping evidence.

### 2.4 Study selection and data extraction

All retrieved records were imported into reference-management software, and duplicates were removed before screening. Two reviewers independently screened titles and abstracts, followed by full-text assessment of potentially eligible publications. Disagreements were resolved through discussion, and a third reviewer was consulted when consensus could not be reached. Reasons for exclusion at the full-text stage were recorded.

Data were independently extracted by two reviewers using a predefined standardized form. Extracted information included first author, publication year, journal, review type, primary-study design, number of included primary studies, number of participants, search date, population characteristics, stroke stage when reported, robot-assisted intervention type, device category and training setting, comparator, balance-related outcomes, and main balance-related findings. Stroke stage was recorded using the terminology of each review and summarized as acute, subacute, chronic, mixed, or not clearly reported. Outcome-level data were also extracted, including outcome domain, outcome measure, number of contributing studies or randomized controlled trials, effect estimate, 95% confidence interval, certainty of evidence, and direction of conclusion. When review-level participant totals were not directly reported, Supplementary material were checked. For multi-arm trials, shared control groups were not double-counted when recalculating participant totals.

### 2.5 Methodological quality assessment

The methodological quality of included systematic reviews was assessed using A MeaSurement Tool to Assess Systematic Reviews 2 (AMSTAR-2) (33). Each item was judged as yes, partial yes, no, or not applicable. The overall confidence in the results of each review was rated as high, moderate, low, or critically low according to AMSTAR-2 guidance. Particular attention was paid to critical domains, including protocol registration, adequacy of the literature search, justification for excluding individual studies, risk-of-bias assessment of included primary studies, appropriateness of meta-analytic methods, consideration of risk of bias when interpreting results, and assessment of publication bias.

### 2.6 PRISMA reporting completeness

Reporting completeness was summarized using selected items from PRISMA 2020 (32). The assessment covered key reporting domains, including title, abstract, rationale, objectives, eligibility criteria, information sources, search strategy, selection process, data extraction process, risk-of-bias methods, synthesis methods, study selection flow, study characteristics, risk-of-bias results, results of syntheses, certainty assessment, protocol registration, funding, and conflicts of interest. PRISMA reporting was summarized descriptively and was not treated as a methodological quality score.

### 2.7 Risk of bias assessment

The risk of bias of included reviews was assessed using the Risk of Bias in Systematic Reviews (ROBIS) tool. ROBIS evaluates concerns across four domains: study eligibility criteria, identification and selection of studies, data collection and study appraisal, and synthesis and findings. The first four domains were judged as low, unclear, or high concern, and an overall risk-of-bias judgment was then assigned as low, unclear, or high risk. ROBIS domains 1–4 were interpreted as concerns, whereas the final judgment was interpreted as overall risk of bias in the review.

### 2.8 Certainty of evidence assessment

The certainty of evidence for major balance-related outcome entries was judged using the Grading of Recommendations Assessment, Development and Evaluation (GRADE) approach. Certainty was rated as high, moderate, low, or very low based on review-level information, including risk of bias, inconsistency, indirectness, imprecision, and publication bias. The GRADE assessment was conducted at the outcome level. Individual primary randomized controlled trial data were not reanalyzed.

### 2.9 Primary-study overlap

Primary-study overlap across included reviews was quantified using the corrected covered area (CCA). CCA was calculated as follows: CCA = (N-r)/(r × c-r), where N represents the total number of primary-study occurrences across reviews, r represents the number of unique primary studies, and c represents the number of reviews included in the matrix. CCA values were interpreted as slight overlap (0–5%), moderate overlap (6–10%), high overlap (11–15%), and very high overlap (>15%).

Two CCA approaches were used. In the harmonized main analysis, obvious primary-study name variants, author-name variants, and same-trial records were standardized. In the conservative exact matching analysis, only exactly matched primary-study identifiers were treated as the same trial. CCA was calculated for overall balance-related outcomes and for major outcome domains, including BBS, TUG/TUGT, FMB/FMA-balance, and postural sway/COP or instrumented postural-control outcomes.

### 2.10 Data synthesis

A narrative synthesis was conducted to summarize the characteristics, methodological quality, reporting completeness, risk of bias, certainty of evidence, and primary-study overlap of the included reviews. Evidence mapping was used to visually summarize the distribution of review-level evidence according to robotic approach and balance-related outcome domain (31). Robotic interventions were grouped primarily according to the device categories and training modes reported in each review; mechanistic characteristics were considered in narrative comparisons when reported. Pooled effect estimates were extracted when available, but no new meta-analysis of primary trials was performed. Findings from reviews sharing primary studies were interpreted in conjunction with the CCA and were not treated as independent replications. Stroke-stage-specific effects were summarized narratively when reported.

## 3 Results

### 3.1 Study selection

**Figure 1:**
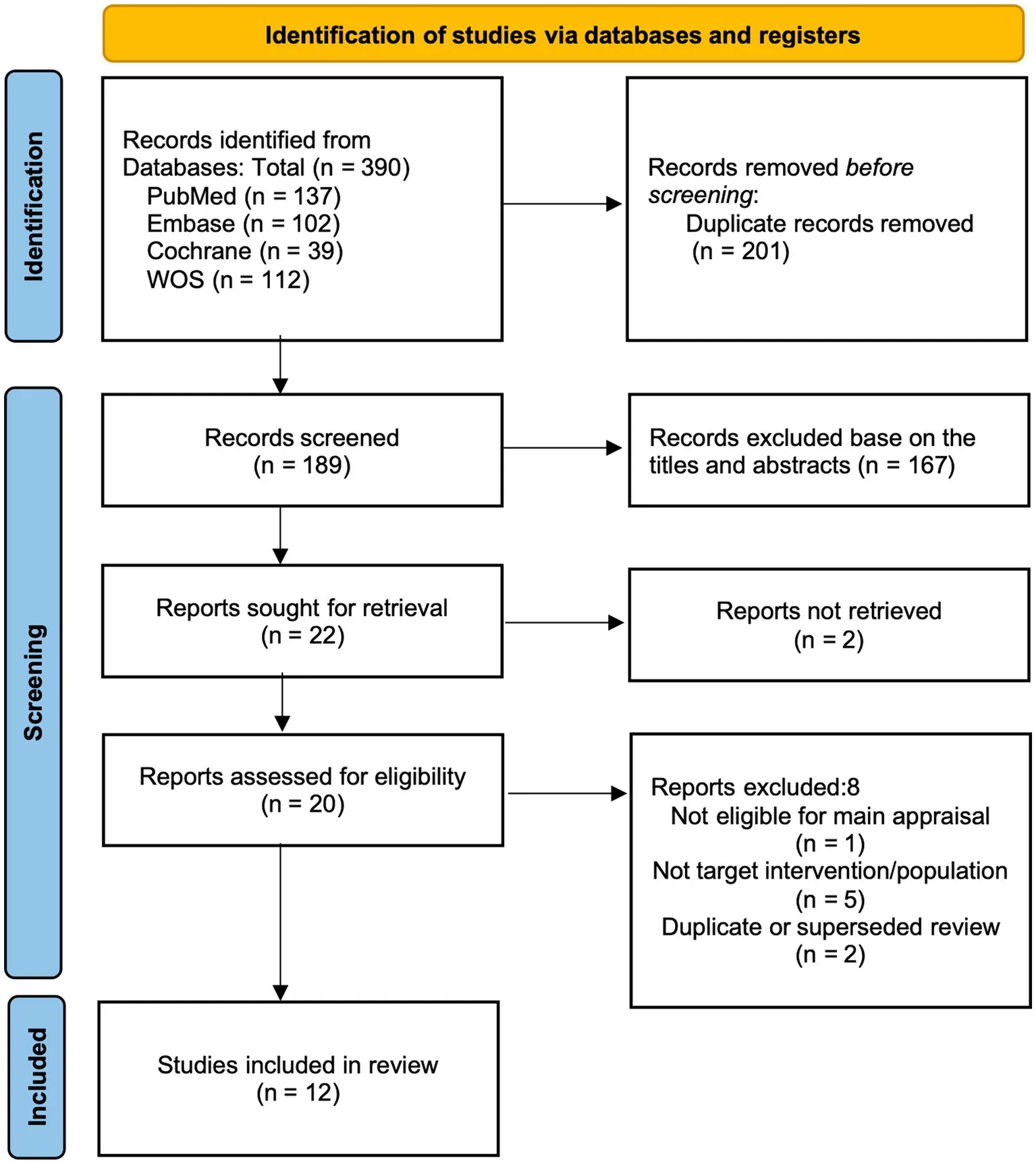
PRISMA 2020 flow diagram of study selection.

The study selection process is shown in Figure 1. A total of 390 records were identified through database searching, including 137 from PubMed, 102 from Embase, 39 from the Cochrane Library, and 112 from Web of Science. After removal of 201 duplicate records, 189 records were screened by title and abstract. Of these, 167 records were excluded. Twenty-two reports were sought for retrieval. Two reports could not be retrieved, and 20 reports were assessed for eligibility. Eight reports were excluded at the full-text stage: one was not eligible for the main appraisal, five did not meet the target intervention or population criteria, and two were duplicate or superseded reviews. Finally, 12 systematic reviews and meta-analyses (26, 27, 39–48) were included in the main appraisal.

### 3.2 Characteristics of included reviews

The characteristics of the included reviews are summarized in Table 1. The 12 included reviews were published between 2014 and 2026. Most reviews were systematic reviews with meta-analysis, one was a systematic review without meta-analysis, and one was a systematic review with network meta-analysis. The primary studies included in these reviews were mainly randomized controlled trials, whereas the earliest review also included controlled and pre-experimental studies.

**Table 1:** Characteristics of the included systematic reviews and meta-analyses.

Study	Year	Journal	Review type	Primary study design	No. of primary studies	No. of participants	Search date	Population	Robot scope	Comparison	Balance outcomes	Main finding
Swinnen et al. (45)	2014	Topics in Stroke Rehabilitation	Systematic review	Controlled and pre-experimental studies	9	359	Inception to 2013	Adult stroke patients	Mixed robotic devices	Other gait rehabilitation or no control	TUG; BBS; postural sway/COP; Tinetti/POMA	Within-group balance improvement after RAGT; between-group superiority unclear.
Zheng et al. (48)	2019	International Journal of Nursing Studies	Systematic review and meta-analysis	RCTs	31	1249	Inception to March 2018	Stroke patients	Robot-assisted gait training	Routine/conventional rehabilitation or non-robot therapy	TUG; BBS; FMB; FRT/SFRT; postural sway/COP; Tinetti/POMA; TIS; ABC	BBS and FMB favored robot-assisted therapy; TUG was not significant.
Wang et al. (46)	2021	Journal of Rehabilitation Medicine	Systematic review and meta-analysis	RCTs	19	517	Inception to 17 January 2020	Patients with stroke	Robot-assisted gait training	Conventional therapy	TUG; BBS; postural sway/COP; Tinetti/POMA; TIS	BBS favored robot-assisted therapy; clearer in acute/subacute and exoskeleton subgroups.
Baronchelli et al. (39)	2021	Frontiers in Neurology	Systematic review and meta-analysis	RCTs	13	445	January 1989 to August 2020	Subacute or chronic stroke patients	Lokomat	Conventional physiotherapy and/or treadmill or other rehabilitation	TUG; BBS; PASS; Tinetti/POMA; RMI; RMA; SPPB	TUG and RMI favored Lokomat; BBS was inconclusive.
Lorusso et al. (43)	2022	Brain Sciences	Systematic review and meta-analysis	RCTs	9	273	Inception to 30 November 2021	Stroke survivors	Overground RAGT	Conventional therapy or o-RAGT with/without conventional therapy	TUG; BBS; FRT; postural sway/COP	BBS meta-analysis did not show superiority over conventional therapy.
Loro et al. (42)	2023	Brain Sciences	Systematic review and meta-analysis	RCTs	18	630	Up to June 2022	Post-stroke survivors	Lower limb rehabilitation robot	Conventional treatment	TUG; BBS	BBS favored RAGT; TUG favored RAGT but was not statistically significant.
Yoo and Lee (47)	2023	Brain and NeuroRehabilitation	Systematic review and meta-analysis	RCTs	12	438	NR	Stroke survivors	Robot-assisted gait training	Conventional therapy	BBS	RAGT was superior to conventional therapy for balance.
Chen et al. (27)	2024	European Journal of Physical and Rehabilitation Medicine	Systematic review and meta-analysis	RCTs	27	1167	Until September 2023	Stroke survivors with lower limb motor dysfunction	Exoskeleton robot	Conventional training or treadmill walking	BBS	BBS was a secondary outcome and did not show statistically clear superiority in the displayed analysis.
Huang et al. (41)	2024	Neurological Sciences	Systematic review and network meta-analysis	RCTs	21	822	Inception to January 2024	Stroke patients	Robot-assisted gait training	Conventional rehabilitation; indirect comparisons between prescription groups	BBS	Network meta-analysis; BBS favored RAGT, but balance was a secondary outcome rather than the main dose-target outcome.
Hao et al. (40)	2025	Systematic Reviews	Systematic review and meta-analysis	RCTs	41	3279	Inception to April 2024	Stroke patients with hemiplegia	Mixed robotic devices	Conventional rehabilitation	BBS	BBS favored robot therapy, both alone RT and CR plus RT comparisons; heterogeneity was high.
Wang et al. (26)	2025	Frontiers in Human Neuroscience	Systematic review and meta-analysis	RCTs	12	651	Inception to February 2024	Adult stroke patients with lower-extremity dysfunction	Lower limb rehabilitation robot	Traditional physical therapy/conventional rehabilitation	TUGT; TUG; BBS	BBS favored robot-assisted training; TUGT was not significant.
Lou et al. (44)	2026	Frontiers in Neurology	Systematic review and meta-analysis	RCTs	16	789	Inception to December 2025	Adults with ischemic or hemorrhagic stroke and lower limb dysfunction/balance impairment	End-effector robot	Conventional rehabilitation without robot training	BBS	BBS favored end-effector robot training, with high heterogeneity.

The no. of primary studies column uses numeric counts only; primary-study design is shown separately. No. of participants is reported at review level unless otherwise specified. For broader reviews, numbers refer to the balance-related robotic gait training subset when the full review-level total was not applicable to the balance evidence. Wang et al. (46) reports 517 participants in the BBS quantitative synthesis. Yoo and Lee (47) reports 438 participants in the RAGT balance meta-analysis; the 12-study count refers to the RAGT balance subset. For Loro et al. (42), the total number of participants was recalculated from Supplementary Table S1 by counting each unique primary trial once and avoiding double counting of shared control groups in multi-arm trials. NR, not reported. BBS, Berg Balance Scale; COP, center of pressure; FMB, Fugl-Meyer balance; RAGT, robot-assisted gait training; RT, robot therapy; TUG, Timed Up and Go; TUGT, Timed Up and Go Test. Balance as secondary outcome: Chen et al. (27) and Huang et al. (41).

The number of included primary studies ranged from 9 to 41 across reviews. The included reviews evaluated robot-assisted rehabilitation for balance-related outcomes in stroke survivors. Robot-assisted interventions included general robot-assisted gait training, Lokomat, overground robot-assisted gait training, end-effector robots, exoskeleton robots, lower-limb rehabilitation robots, and mixed robotic devices. Comparators mainly included conventional rehabilitation, conventional physiotherapy, non-robotic gait training, treadmill walking, or other non-robotic rehabilitation approaches.

BBS was the most frequently reported balance outcome. Other outcomes included TUG or TUGT, FMB, PASS, FRT or SFRT, Tinetti/POMA, TIS, ABC, RMI, RMA, SPPB, postural sway, and COP-based instrumented postural-control measures. Several reviews directly focused on balance rehabilitation, whereas others primarily evaluated lower-limb function, gait ability, or robotic gait training and reported balance as a secondary outcome.

**Figure 2:**
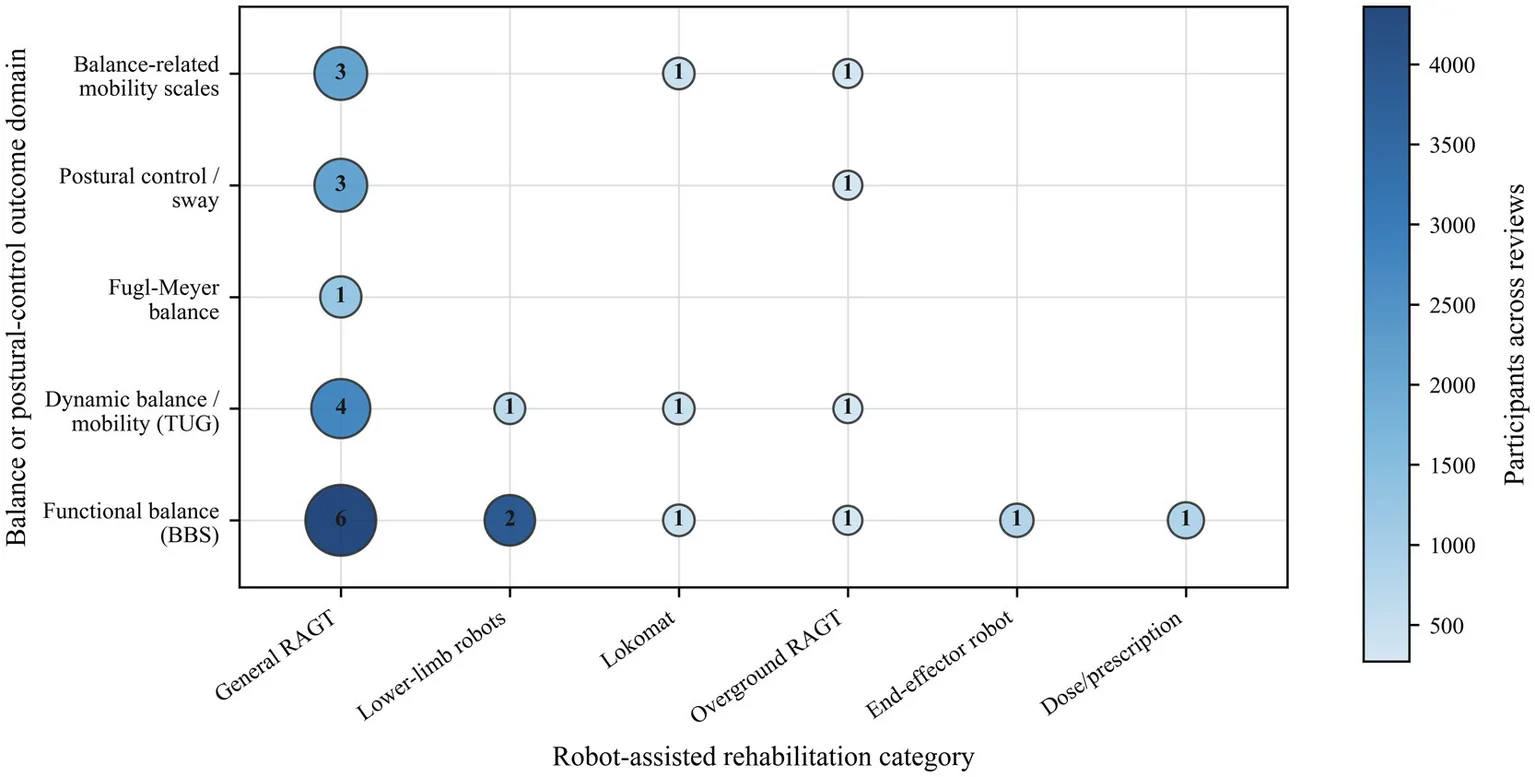
Evidence map of included reviews according to robot type and balance-related outcomes. One bubble per cell; size = total primary studies, color = total participants, number = SR/MA count. Bubble size represents the summed number of primary studies reported by reviews in each cell; color represents summed participants. Because this is an umbrella, values are review-level mapped counts and may include overlapping primary trials.

The evidence map is shown in Figure 2. The map indicated that review-level evidence was concentrated in general robot-assisted gait training and lower-limb robotic rehabilitation, with BBS and TUG/TUGT being the most frequently represented outcome domains. Evidence for FMB/FMA-balance and postural sway/COP was less frequently reported.

### 3.3 Methodological quality assessed by AMSTAR-2

The AMSTAR-2 assessment is presented in Table 2. Overall, two reviews were rated as critically low methodological quality, and 10 reviews were rated as low methodological quality. No review was rated as high or moderate quality. The main methodological limitations were lack of a complete list of excluded studies with reasons, incomplete reporting of funding sources for included primary studies, and insufficient consideration of how risk of bias in primary studies affected the synthesis and interpretation of results.

**Table 2:** AMSTAR-2 assessment of methodological quality in the included systematic reviews.

Study	Item 1	Item 2*	Item 3	Item 4*	Item 5	Item 6	Item 7*	Item 8	Item 9*	Item 10	Item 11*	Item 12	Item 13*	Item 14	Item 15*	Item 16	Overall
Swinnen et al. (45)	Y	N	Y	PY	PY	PY	N	Y	PY	N	NA	PY	PY	Y	N	PY	Critically low
Zheng et al. (48)	Y	PY	Y	Y	Y	Y	N	Y	Y	N	Y	PY	PY	Y	Y	Y	Low
Wang et al. (46)	Y	Y	Y	Y	Y	Y	N	Y	Y	N	Y	Y	Y	Y	Y	Y	Low
Baronchelli et al. (39)	Y	Y	Y	Y	Y	Y	N	Y	Y	N	Y	PY	PY	Y	PY	Y	Low
Lorusso et al. (43)	Y	N	Y	PY	Y	Y	N	Y	Y	N	Y	PY	PY	Y	NA	Y	Critically low
Loro et al. (42)	Y	Y	Y	PY	Y	Y	N	Y	Y	N	Y	PY	Y	Y	Y	Y	Low
Yoo and Lee (47)	Y	PY	Y	PY	PY	PY	N	Y	Y	N	Y	Y	Y	Y	Y	Y	Low
Chen et al. (27)	Y	Y	Y	PY	Y	Y	N	Y	Y	N	Y	PY	Y	Y	Y	Y	Low
Huang et al. (41)	Y	Y	Y	Y	Y	Y	N	Y	Y	N	Y	PY	PY	Y	PY	Y	Low
Hao et al. (40)	Y	Y	Y	Y	Y	Y	N	Y	Y	N	Y	PY	PY	Y	Y	Y	Low
Wang et al. (26)	Y	Y	Y	Y	Y	Y	N	Y	Y	N	Y	PY	Y	Y	NA	Y	Low
Lou et al. (44)	Y	Y	Y	Y	Y	Y	N	Y	Y	N	Y	PY	Y	Y	Y	Y	Low

Y = Yes; PY = Partial yes; N = No; NA = Not applicable. Critical AMSTAR-2 domains are marked with an asterisk. AMSTAR-2 assesses methodological quality and is not equivalent to certainty of clinical evidence.

Several reviews also had limitations in protocol registration, comprehensiveness of the search strategy, assessment or interpretation of publication bias, and explanation of heterogeneity. Older reviews and reviews with less complete reporting tended to receive lower ratings. These findings indicate that the methodological credibility of existing review-level evidence is limited.

### 3.4 PRISMA 2020 reporting completeness

PRISMA 2020 reporting completeness is summarized in Table 3. Recent reviews generally reported key items, including objectives, eligibility criteria, information sources, search strategies, selection process, synthesis methods, study selection flow diagrams, study characteristics, and results of syntheses. However, protocol registration, certainty assessment, funding of included primary studies, and conflicts-of-interest information were inconsistently reported.

**Table 3:** PRISMA 2020 reporting completeness of the included systematic reviews.

Study	Title	Abstract	Rationale	Objectives	Eligibility	Sources	Search	Selection	Extraction	RoB methods	Synthesis	Flow	Characteristics	RoB results	Results	Certainty	Protocol	Funding/COI
Swinnen et al. (45)	N	N	PY	Y	Y	PY	PY	Y	PY	PY	PY	Y	Y	PY	PY	N	N	PY
Zheng et al. (48)	Y	Y	Y	Y	Y	Y	Y	Y	Y	Y	Y	Y	Y	Y	Y	N	N	Y
Wang et al. (46)	Y	Y	Y	Y	Y	Y	Y	Y	Y	Y	Y	Y	Y	Y	Y	N	Y	Y
Baronchelli et al. (39)	Y	Y	Y	Y	Y	Y	Y	Y	Y	Y	Y	Y	Y	Y	Y	N	Y	Y
Lorusso et al. (43)	Y	Y	Y	Y	Y	Y	Y	Y	Y	Y	Y	Y	Y	Y	Y	N	N	Y
Loro et al. (42)	Y	Y	Y	Y	Y	Y	Y	Y	Y	Y	Y	Y	Y	Y	Y	N	Y	Y
Yoo and Lee (47)	Y	Y	Y	Y	Y	PY	PY	Y	PY	Y	Y	Y	Y	Y	Y	Y	N	Y
Chen et al. (27)	Y	Y	Y	Y	Y	Y	Y	Y	Y	Y	Y	Y	Y	Y	Y	N	Y	Y
Huang et al. (41)	Y	Y	Y	Y	Y	Y	Y	Y	Y	Y	Y	Y	Y	Y	Y	N	Y	Y
Hao et al. (40)	Y	Y	Y	Y	Y	Y	Y	Y	Y	Y	Y	Y	Y	Y	Y	PY	Y	Y
Wang et al. (26)	Y	Y	Y	Y	Y	Y	Y	Y	Y	Y	Y	Y	Y	Y	Y	PY	Y	Y
Lou et al. (44)	Y	Y	Y	Y	Y	Y	Y	Y	Y	Y	Y	Y	Y	Y	Y	Y	Y	Y

Y, yes; PY, partial yes; N, no; NA, not applicable. PRISMA reporting was summarized descriptively and was not used as a methodological quality score.

The earliest review showed less complete reporting across several items, including title, abstract, search strategy, data extraction process, risk-of-bias methods, and certainty assessment. Overall, reporting completeness improved in more recent reviews, but important gaps remained, particularly in protocol availability and certainty-of-evidence reporting. PRISMA reporting was interpreted descriptively and was not used as a methodological quality score.

### 3.5 Risk of bias assessed by ROBIS

**Figure 3:**
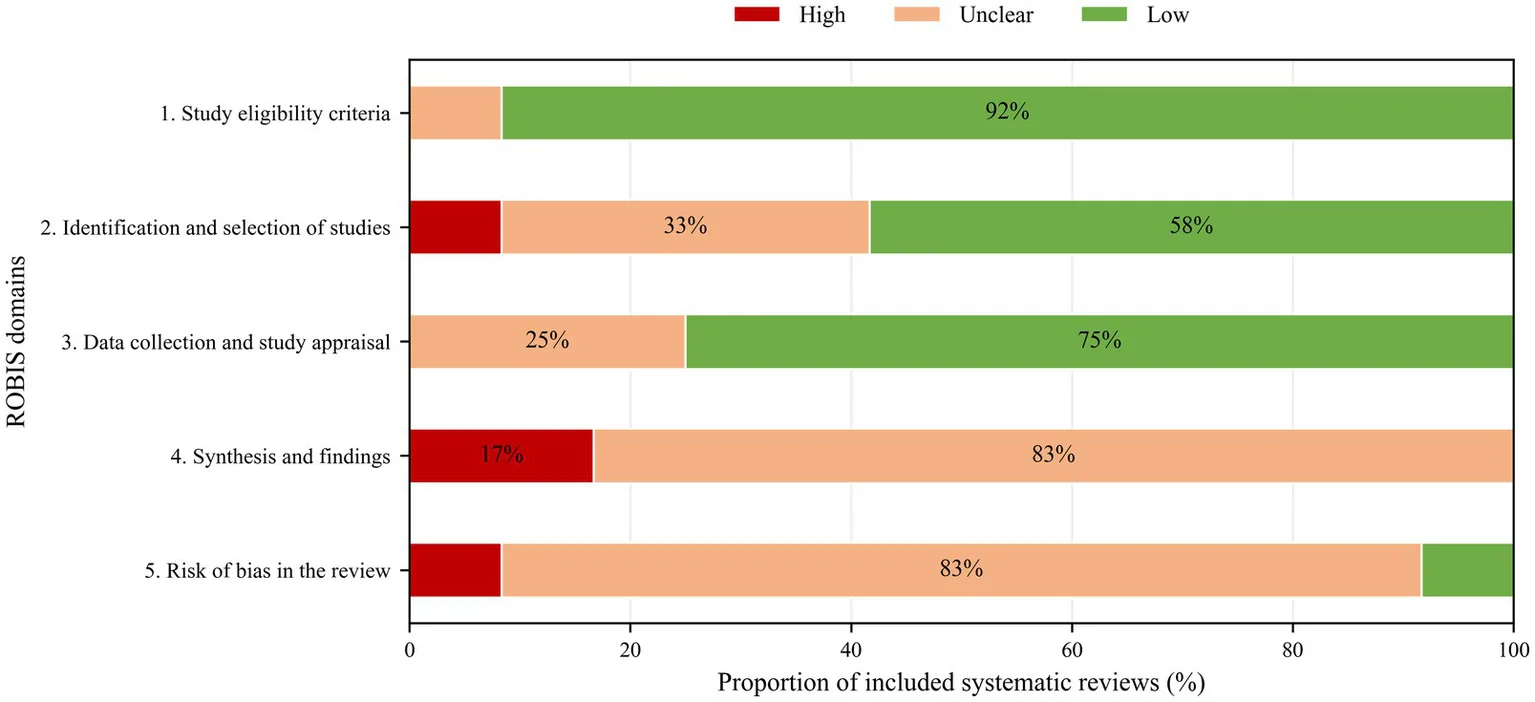
Graphical presentation of ROBIS judgments across included systematic reviews.

The ROBIS assessment is presented in Table 4, and the graphical summary is shown in Figure 3. One review was judged as having an overall high risk of bias, whereas the remaining reviews were judged as having unclear overall risk of bias. No review was judged as having low overall risk of bias.

**Table 4:** ROBIS risk-of-bias assessment of the included systematic reviews.

Study	D1 eligibility criteria	D2 identification/selection	D3 collection/appraisal	D4 synthesis/findings	Overall ROBIS	Rationale
Swinnen et al. (45)	Low concern	High concern	Unclear concern	Unclear concern	High risk	Older review with limited databases and mixed study designs.
Zheng et al. (48)	Low concern	Low concern	Unclear concern	Unclear concern	Unclear risk	Comprehensive database search, but heterogeneity and possible publication bias remain.
Wang et al. (46)	Low concern	Low concern	Low concern	Unclear concern	Unclear risk	Registered protocol and appropriate synthesis; some heterogeneity and small subgroups.
Baronchelli et al. (39)	Low concern	Low concern	Low concern	Unclear concern	Unclear risk	Device-specific question is clear; synthesis limited by few and heterogeneous studies.
Lorusso et al. (43)	Low concern	Unclear concern	Unclear concern	Unclear concern	Unclear risk	Specific overground scope; small number of studies and limited quantitative synthesis.
Loro et al. (42)	Low concern	Unclear concern	Low concern	Unclear concern	Unclear risk	Clear balance-focused question; high clinical and protocol heterogeneity.
Yoo and Lee (47)	Unclear concern	Unclear concern	Low concern	Unclear concern	Unclear risk	Broad RAT question; balance evidence is a RAGT subgroup.
Chen et al. (27)	Low concern	Unclear concern	Low concern	Unclear concern	Unclear risk	Gait-focused review with BBS as secondary outcome; limited databases.
Huang et al. (41)	Low concern	Low concern	Low concern	Unclear concern	Unclear risk	Network methods useful for dose but balance is not the main network target.
Hao et al. (40)	Low concern	Low concern	Low concern	High concern	Unclear risk	Large evidence base but BBS heterogeneity was very high.
Wang et al. (46)	Low concern	Low concern	Low concern	Unclear concern	Unclear risk	Recent focused review; subgroup and TUGT evidence are limited.
Lou et al. (44)	Low concern	Low concern	Low concern	High concern	Unclear risk	End-effector scope is clear, but BBS heterogeneity is high.

ROBIS judgments were made at review level. D1, study eligibility criteria; D2, identification and selection of studies; D3, data collection and study appraisal; D4, synthesis and findings.

Concerns were most evident in the synthesis and findings domain. These concerns were mainly related to substantial heterogeneity in balance-related analyses, limited ability to fully explain heterogeneity, small numbers of contributing primary studies in some outcome domains, and broad review questions in which balance evidence was derived from robotic gait training subgroups. Eligibility criteria were generally clear in most reviews, while identification and selection of studies showed greater concerns in older or less comprehensively reported reviews.

### 3.6 Summary of balance-related outcomes and certainty of evidence

Major balance-related outcome findings and certainty of evidence are summarized in Table 5. Overall, the evidence suggested that robot-assisted rehabilitation may improve BBS scores compared with conventional or non-robotic rehabilitation, but the certainty of evidence was generally low or very low. Evidence for TUG/TUGT, FMB, RMI, and instrumented postural-control outcomes was less consistent and more uncertain. Compared with BBS, these outcomes were generally reported in fewer reviews or supported by fewer primary studies, and their findings showed greater heterogeneity. Although favorable findings were reported for different robotic approaches, no single approach was consistently superior across balance-related outcomes or across reviews.

**Table 5:** Summary of major balance-related outcomes and certainty of evidence.

Study	Outcome domain	Outcome	No. of studies/RCTs	Effect estimate	95% CI	Certainty	Direction/conclusion
Zheng et al. (48)	Balance	BBS	23	MD 4.64	3.22–6.06	Low	Favored robot-assisted therapy
	Balance	FMB	3	MD 3.57	2.81–4.34	Very low	Favored robot-assisted therapy
	Dynamic balance/mobility	TUG	3	MD -5.60	−13.69 to 2.49	Very low	Not statistically significant
Wang et al. (46)	Balance	BBS	13	WMD 3.58	1.89–5.28	Low	Favored robot-assisted therapy
Baronchelli et al. (39)	Balance	BBS	9	MD 0.17	−0.26 to 0.60	Very low	Not statistically significant
	Dynamic balance/mobility	TUG	5	MD -3.40	−4.35 to −2.44	Very low	Favored Lokomat
	Balance-related mobility	RMI	4	MD 0.40	0.26–0.55	Very low	Favored Lokomat
Lorusso et al. (43)	Balance	BBS	3	MD -0.01	−3.24 to 3.26	Very low	Not statistically significant
Loro et al. (42)	Balance	BBS	15	pMD 2.17	0.79–3.55	Low	Favored RAGT
	Dynamic balance/mobility	TUG	9	pMD -0.62	−3.66 to 2.43	Very low	Not statistically significant
Yoo and Lee (47)	Balance	Balance outcome, mainly BBS-based	12	MD 2.47	0.41–4.53	Low	Favored RAGT
Chen et al. (27)	Balance	BBS	9	SMD -0.33	−1.03 to 0.37	Very low	Not statistically significant
Huang et al. (41)	Balance	BBS	7	MD 3.63	2.46–4.80	Low	Favored RAGT; secondary outcome
Hao et al. (40)	Balance	BBS; RT alone vs. CR	4	MD 12.22	3.54–20.91	Very low	Favored RT; heterogeneity was very high
	Balance	BBS; CR plus RT vs. CR	22	MD 10.64	8.04–13.25	Very low	Favored CR plus RT; heterogeneity was very high
Wang et al. (26)	Balance	BBS	6	MD 6.98	3.06–10.98	Low	Favored robot-assisted training
	Dynamic balance/mobility	TUGT	3	MD -1.05	−4.90 to 2.80	Very low	Not statistically significant
Lou et al. (44)	Balance	BBS	11	MD 5.24	1.10–9.38	Very low	Favored end-effector robot; heterogeneity was very high

Values refer to the number of primary studies contributing to each outcome. Swinnen et al. (45) was not included in this table because no pooled effect estimate was available. Certainty was judged in the present overview using review-level information; individual primary RCT data were not reanalyzed. For TUG/TUGT, negative values indicate shorter completion time and therefore improvement. CR, conventional rehabilitation; FMB, Fugl-Meyer balance; RAGT, robot-assisted gait training; RMI, Rivermead Mobility Index; RT, robot therapy; TUG, Timed Up and Go; TUGT, Timed Up and Go Test.

For BBS, several reviews reported statistically favorable effects of robot-assisted rehabilitation. Zheng et al. reported a mean difference of 4.64, Wang et al. reported a weighted mean difference of 3.58, Loro et al. reported a pooled mean difference of 2.17, Huang et al. reported a mean difference of 3.63, Wang et al. reported a mean difference of 6.98, and Lou et al. reported a mean difference of 5.24 (26, 42, 44, 46, 48, 49). In the 2021 review by Wang et al. (46), subgroup analyses showed a significant BBS benefit in acute or subacute stroke (WMD 5.40, 95% CI 3.94–6.86), whereas the effect in chronic stroke was not statistically significant (WMD 1.61, 95% CI − 0.02 to 3.25). Stage-specific estimates were otherwise rarely reported across the included reviews. However, Chen et al. did not find a statistically clear BBS advantage, and Lorusso et al. reported no superiority of overground robot-assisted gait training over conventional therapy for BBS (27, 43). The certainty of BBS evidence ranged from low to very low.

Evidence for TUG or TUGT was less consistent. Baronchelli et al. reported that Lokomat training improved TUG compared with conventional therapy, whereas Zheng et al., Loro et al., and Wang et al. did not identify statistically significant improvements in TUG or TUGT (26, 39, 42, 48). The certainty of evidence for TUG/TUGT was judged as very low. Evidence for FMB and RMI was also limited and rated as very low. Instrumented postural-control outcomes, including postural sway and COP-based measures, were reported less frequently and were mainly descriptive or based on a small number of primary studies.

Overall, no major balance-related outcome entry was rated as high certainty. Most outcome entries were rated as low or very low certainty, reflecting methodological limitations, inconsistency, imprecision, heterogeneity, and possible publication bias.

### 3.7 Primary-study overlap assessed by CCA

**Figure 4:**
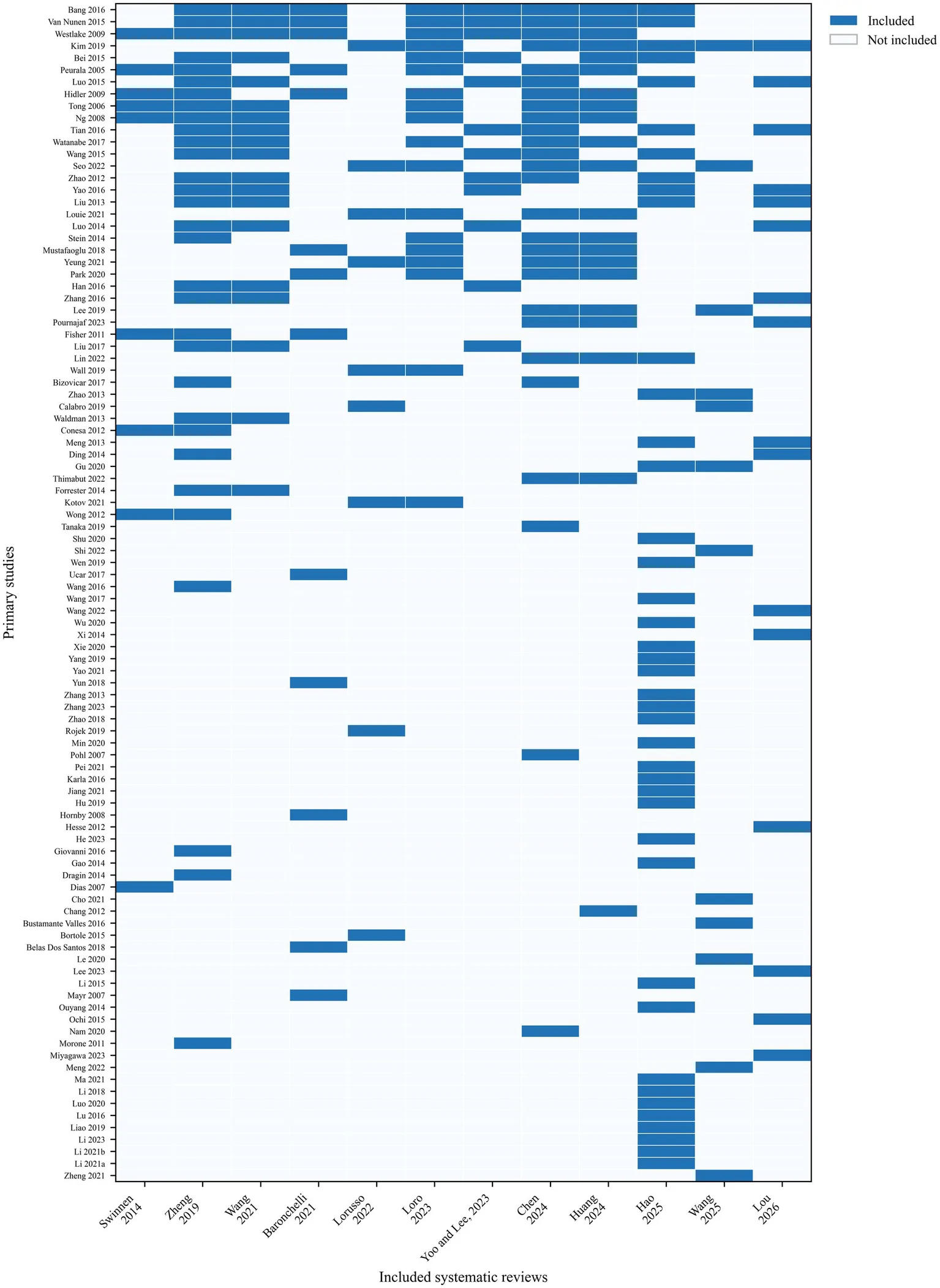
Corrected covered area overlap matrix of primary studies. CCA, corrected covered area (overall CCA = 0.121). Matrix is based on working extraction from review tables/reference lists and should be verified before final submission.

The degree of primary-study overlap across included reviews is presented in Table 6, and the overlap matrix is shown in Figure 4. In the harmonized main analysis, the overall balance-related evidence showed high overlap, with a CCA of 12.06%. The BBS-reporting evidence domain also showed high overlap, with a CCA of 11.61%. Similarly, the TUG/TUGT-reporting evidence domain showed high overlap, with a CCA of 11.76%. The postural sway/COP or instrumented postural-control domain showed very high overlap, with a CCA of 44.44%, although this estimate was based on only two contributing reviews. FMB/FMA-balance evidence was contributed by only one review and was therefore not suitable for CCA calculation.

**Table 6:** Corrected covered area of primary-study overlap across included systematic reviews.

Analysis	Topic	*N*	*r*	*c*	N-r	r × c-r	CCA (%)	Overlap
Harmonized main analysis	Overall balance-related outcomes	228	98	12	130	1,078	12.06%	High overlap
Harmonized main analysis	BBS-reporting balance evidence	134	62	11	72	620	11.61%	High overlap
Harmonized main analysis	TUG/TUGT-reporting balance evidence	23	17	4	6	51	11.76%	High overlap
Harmonized main analysis	FMB/FMA-balance evidence	3	3	1	0	NA	NA	NA
Harmonized main analysis	Postural sway/COP or instrumented postural control	13	9	2	4	9	44.44%	Very high overlap
Conservative exact matching	Overall balance-related outcomes	228	104	12	124	1,144	10.84%	High overlap
Conservative exact matching	BBS-reporting balance evidence	134	70	11	64	700	9.14%	Moderate overlap
Conservative exact matching	TUG/TUGT-reporting balance evidence	23	18	4	5	54	9.26%	Moderate overlap
Conservative exact matching	FMB/FMA-balance evidence	3	3	1	0	NA	NA	NA
Conservative exact matching	Postural sway/COP or instrumented postural control	13	10	2	3	10	30.00%	Very high overlap

CCA = (N-r)/(r × c-r), where N is the number of included primary-study occurrences, r is the number of unique primary studies, and c is the number of reviews included in that analysis. The harmonized main analysis standardized obvious primary-study name variants, author-name variants, and same-trial records. Conservative exact matching retained only exactly matched primary-study identifiers and therefore gives a lower-bound overlap estimate. Domain rows were calculated from the currently extractable outcome-domain evidence map and should be rechecked against final primary-study reference harmonization before submission. NA indicates that CCA is not mathematically meaningful when only one review contributes to that domain. Overlap interpretation: slight = 0–5%; moderate = 6–10%; high = 11–15%; very high >15%.

In the conservative exact matching analysis, the overall balance-related evidence still showed high overlap, with a CCA of 10.84%. BBS-reporting evidence and TUG/TUGT-reporting evidence showed moderate overlap, with CCA values of 9.14 and 9.26%, respectively. Postural sway/COP or instrumented postural-control evidence remained characterized by very high overlap. These findings indicate that several reviews summarized overlapping sets of primary studies, particularly for BBS and TUG/TUGT outcomes. Therefore, the review-level evidence should not be interpreted as fully independent.

### 3.8 Overall evidence profile

Taken together, the current review-level evidence suggests that robot-assisted rehabilitation may have potential benefits for selected balance-related outcomes after stroke, especially BBS. However, the evidence base is limited by low or critically low methodological quality of existing reviews, unclear or high risk of bias, low or very low certainty of evidence, substantial heterogeneity, and high primary-study overlap. The findings for dynamic balance and mobility outcomes, particularly TUG/TUGT, remain uncertain. Therefore, the overall evidence supports a cautious conclusion that robot-assisted rehabilitation may be useful as an adjunct to comprehensive post-stroke rehabilitation, but stronger evidence is needed to define its effects on balance function and postural control.

## 4 Discussion

### 4.1 Principal findings

This umbrella review summarized evidence from 12 systematic reviews and meta-analyses evaluating robot-assisted rehabilitation for balance function and postural control after stroke. The included reviews covered robot-assisted gait training, Lokomat, overground robot-assisted gait training, end-effector robots, exoskeleton robots, lower-limb rehabilitation robots, and mixed robotic devices. Overall, robot-assisted rehabilitation showed favorable signals for selected balance-related outcomes, particularly the Berg Balance Scale (BBS). However, the certainty of evidence was generally low or very low, and findings for Timed Up and Go (TUG/TUGT), Fugl-Meyer balance (FMB), Rivermead Mobility Index (RMI), and instrumented postural-control outcomes were less consistent.

These findings should be interpreted in the clinical context of post-stroke balance impairment. Balance dysfunction after stroke is highly prevalent and strongly associated with ADL dependence, mobility limitation, fear of falling, and reduced quality of life (1, 2, 6). Dynamic balance has been identified as an important predictor of ADL independence among stroke survivors with a history of falls, and longitudinal evidence suggests that poorer baseline lower-limb function, transfer ability, and older age may predict higher subsequent fall risk and functional decline (5, 6). Therefore, even modest improvements in balance-related function may have clinical relevance when they translate into better transfer ability, walking safety, and daily independence.

### 4.2 Why BBS and TUG/TUGT showed different evidence patterns

In this umbrella review, BBS showed a more consistent favorable signal than TUG/TUGT. This difference may partly reflect the different constructs measured by these outcomes. BBS mainly assesses functional balance during structured tasks such as sitting, standing, transfers, reaching, turning, and maintaining position under standardized conditions (50). It is useful for evaluating basic functional balance and standing control, but it may be less sensitive to complex dynamic balance, gait adaptability, dual-task demands, and higher-level mobility challenges (51). By contrast, TUG/TUGT requires sequential performance of sit-to-stand transition, gait initiation, walking, turning, return walking, and sitting down (52). It reflects not only balance but also gait speed, lower-limb coordination, turning control, mobility confidence, and executive control during continuous movement (52, 53).

Robot-assisted gait training may improve repetitive stepping, weight transfer, lower-limb loading, and basic postural stability, which may be sufficient to produce measurable gains in BBS. However, improvement in TUG/TUGT may require broader recovery of walking speed, dynamic balance, turning ability, trunk control, gait adaptability, and confidence under continuous movement demands. This may explain why some reviews reported significant BBS improvement while TUG/TUGT remained statistically non-significant. Clinically, BBS improvement should therefore be viewed as evidence of better functional balance, but not necessarily as proof of improved complex mobility or fall-related dynamic stability.

### 4.3 Possible mechanisms linking robotic rehabilitation to balance recovery

Several mechanisms may explain why robot-assisted rehabilitation can improve selected balance outcomes after stroke. First, lower-limb robotic systems can provide intensive, repetitive, task-oriented, and quantifiable practice under controlled conditions. Such training is consistent with principles of motor learning and experience-dependent neuroplasticity, and may promote lower-limb motor recovery by increasing the amount and quality of sensorimotor input (26, 40, 54). Second, robotic devices may provide structured weight-bearing and stepping practice, which may improve paretic limb loading, interlimb coordination, and postural symmetry. These mechanisms are clinically relevant because many stroke survivors show greater weight bearing on the non-paretic side, reduced support capacity of the paretic lower limb, increased postural sway, and gait asymmetry (10–13).

Third, robotic rehabilitation may enhance sensory-motor integration. Some lower-limb robotic systems provide tactile, visual, auditory, and proprioceptive feedback, helping patients perceive lower-limb movement and spatial position more accurately (49). Impaired sensory reweighting, involving abnormal integration of visual, somatosensory, and vestibular inputs, has been associated with slower walking speed, gait asymmetry, and impaired acceleration ability in chronic stroke (8). By repeatedly coupling movement guidance with afferent feedback, robotic systems may strengthen sensory-motor coupling and improve movement control.

Fourth, robotic training may influence cortical reorganization and corticospinal excitability. Clinical neuroimaging and neurophysiological studies have reported that lower-limb robotic training may be accompanied by increased activation in the affected motor area and bilateral prefrontal cortices, as well as improvements in BBS, FMA-LE, FAC, MBI, and gait parameters (55). Active-mode robotic training may produce greater FMA improvement, higher motor evoked potential amplitude, and stronger activation of the affected motor cortex than passive-mode training, suggesting that active participation may be important for corticospinal drive and cortical plasticity (56). Studies combining robotic training with cognitive strategies such as motor imagery also suggest that high-intensity, quantifiable task practice may engage fronto-motor connectivity (57). Nevertheless, these mechanisms remain plausible explanations rather than definitive proof, because the certainty of clinical evidence in this umbrella review was generally low or very low.

### 4.4 Device heterogeneity and the distinction between gait-centered and balance-specific robotic training

A major reason for inconsistent findings is the heterogeneity of robotic devices and training paradigms. Lower-limb robotic systems can be broadly categorized into exoskeleton-type devices and end-effector-type devices, and they may be used with treadmill-based, body-weight-supported, or overground walking systems (24–26). End-effector devices, such as Gait Trainer or G-EO systems, simulate stance and swing phases through footplate trajectories, whereas exoskeleton devices, such as Lokomat, Walkbot, and HAL, apply torque or guidance directly at lower-limb joints to guide gait toward a predefined physiological pattern (25, 58, 59). These differences may influence postural-control demands, active participation, trunk and pelvic movement, mediolateral stability, and ecological validity.

Most included reviews evaluated robot-assisted gait training rather than balance-specific robotic training. This distinction is important. Gait-centered RAGT may indirectly improve balance by increasing stepping practice, lower-limb loading, and walking-related task exposure, but it may not adequately train multidirectional limits of stability, perturbation responses, anticipatory postural adjustment, trunk control, or reactive balance. Several studies have suggested that balance-oriented interventions, including vestibular rehabilitation, dual-task training, and integrated balance-gait training, can improve BBS, TUG, step length, cadence, and other mobility outcomes more effectively than walking training or conventional rehabilitation alone in selected contexts (21–23). These findings support the need to distinguish robotic gait training from robot-assisted balance training in future trials and evidence syntheses.

### 4.5 Methodological credibility, certainty of evidence, and overlap

The favorable signals observed for BBS should be weighed against methodological limitations. AMSTAR-2 assessment showed that no included review reached high or moderate methodological quality. Common limitations included lack of complete excluded-study lists, insufficient reporting of funding sources for primary trials, and limited consideration of how primary-study risk of bias influenced synthesis and interpretation. ROBIS assessment also identified concerns, particularly in the synthesis and findings domain, mainly because several reviews included heterogeneous devices, protocols, stroke populations, and outcome definitions.

GRADE certainty was low or very low across major balance-related outcomes. BBS evidence was rated as low in several reviews, but evidence for TUG/TUGT, FMB, RMI, and postural sway/COP outcomes was mostly very low. Some BBS findings from larger or recent reviews were also downgraded to very low because of very high heterogeneity. These findings mean that the true effect may differ substantially from the review-level estimates. They also suggest that statistical significance should not be interpreted as high certainty.

Primary-study overlap further limits the independence of review-level evidence. The CCA analysis showed high overlap for overall balance-related outcomes, BBS-reporting evidence, and TUG/TUGT-reporting evidence, and very high overlap for the postural sway/COP domain. This indicates that multiple systematic reviews summarized overlapping sets of primary trials. Therefore, the number of published reviews should not be interpreted as the number of independent evidence sources. The combination of low or very low certainty and high primary-study overlap supports a cautious interpretation of the current evidence.

### 4.6 Clinical implications

The current evidence suggests that robot-assisted rehabilitation may be considered as an adjunctive approach for selected stroke patients with balance impairment, particularly when the goal is to improve functional balance measured by BBS. However, it does not support replacing conventional therapist-guided rehabilitation with robotic training alone. Balance recovery after stroke requires multidimensional intervention targeting lower-limb strength, trunk control, sensory integration, weight transfer, gait adaptability, turning, anticipatory and reactive postural adjustments, and confidence during functional movement.

This interpretation is consistent with evidence showing that targeted balance training, trunk or core stability training, and integrated balance-gait programs can improve balance, mobility, and ADL independence (14–18). Physical rehabilitation programs that include functional task training, especially tasks that challenge both gait and balance, may be particularly important for improving ADL and motor function after stroke (60). Therefore, robotic systems should ideally be embedded within comprehensive rehabilitation programs that combine conventional physiotherapy with balance-specific, functional, and cognitive–motor training, rather than used as isolated gait-repetition devices.

Patient selection may also influence treatment response. Patients with severe gait impairment may benefit from robotic assistance because it allows safe, repetitive, upright practice with reduced therapist burden. Patients with milder deficits may require more challenging dynamic balance tasks, variable environments, turning, dual-task demands, and perturbation-based training. Although Wang et al. (46) reported a statistically significant BBS benefit in acute or subacute but not chronic stroke, stage-specific estimates were otherwise sparse; stroke phase, baseline mobility level, trunk control, sensory deficits, and cognitive status therefore remain plausible modifiers rather than established determinants of response. Current guidelines generally consider lower-limb robotic rehabilitation as an adjunctive option for improving walking and lower-limb motor function, but there is still no consensus regarding optimal timing, frequency, dose, or target population (61).

### 4.7 Strengths and limitations

This umbrella review has several strengths. It focused specifically on balance function and postural control after stroke, a clinically important domain that is often reported as a secondary outcome in robotic gait rehabilitation research. It combined evidence mapping with AMSTAR-2, PRISMA 2020, ROBIS, GRADE, and CCA, allowing the evidence to be interpreted not only according to effect direction but also according to methodological quality, reporting completeness, risk of bias, certainty of evidence, and primary-study overlap. It also separated BBS, TUG/TUGT, FMB, RMI, and instrumented postural-control outcomes rather than drawing a single broad conclusion about balance.

Several limitations should be acknowledged. First, this umbrella review relied on published systematic reviews and meta-analyses; individual primary trials were not reanalyzed. Second, several included reviews primarily focused on gait or lower-limb motor function, and balance was a secondary outcome. Third, the included reviews differed in robot type, comparator, stroke phase, intervention dose, and outcome definition, limiting direct comparability. Fourth, CCA analysis depended on the completeness and accuracy of primary-study lists reported in the included reviews. Fifth, the included reviews did not consistently report major clinical complications, treatment interruptions, achieved rehabilitation dose, or length of stay. Clostridioides difficile infection has been examined specifically in stroke patients in rehabilitation wards, providing a concrete example of an intercurrent clinical event that may complicate recovery pathways (62). Finally, objective postural-control outcomes such as COP and postural sway were less frequently synthesized than BBS and TUG/TUGT, limiting conclusions about instrumented balance outcomes.

### 4.8 Implications for future research

Future trials should clearly distinguish between gait-centered robotic training and robot-assisted balance training. If the intervention is intended to improve balance or postural control, balance outcomes should be prespecified as primary outcomes, and assessments should include both functional scales and objective postural-control measures where possible. Future studies should also report clinically meaningful change, adverse events, major clinical complications, treatment interruptions, achieved treatment dose, treatment adherence, length of stay, stroke phase, baseline mobility level, device type, assistance mode, active participation level, training frequency, session duration, and co-interventions. These variables should inform the development and testing of personalized robotic protocols matched to patient phenotype, impairment severity, and rehabilitation goals.

Future systematic reviews should improve methodological rigor by prospectively registering protocols, using comprehensive searches, providing excluded-study lists with reasons, reporting primary-study funding sources, applying transparent GRADE assessments, and calibrating conclusions according to evidence certainty. Given the high overlap observed in the current umbrella review, future evidence syntheses should also assess primary-study overlap and avoid treating overlapping reviews as independent evidence. More rigorous primary trials and higher-quality systematic reviews are needed to clarify which patients, robotic devices, and training protocols are most likely to improve balance and postural control after stroke.

## 5 Conclusion

Robot-assisted rehabilitation may improve selected balance-related outcomes after stroke, particularly BBS. However, the certainty of evidence is generally low or very low, and evidence for TUG/TUGT, FMB, RMI, and instrumented postural-control outcomes remains uncertain. Existing reviews are limited by methodological weaknesses, heterogeneity across devices and protocols, and overlap of primary trials. Robot-assisted rehabilitation should therefore be considered an adjunct to multidisciplinary post-stroke rehabilitation rather than a replacement for conventional therapy. Future high-quality randomized trials should distinguish robotic gait training from balance-specific robotic training, use standardized and transparently reported protocols, identify which patients, devices, and training parameters are most likely to be effective, and evaluate long-term functional outcomes together with patient-centered measures.

## Data Availability

The original contributions presented in the study are included in the article/Supplementary material, further inquiries can be directed to the corresponding author.
