# Supplementary material for: Robot-assisted rehabilitation for post-stroke balance and postural control: an umbrella review and evidence map of systematic reviews
Source: Front Neurol. 2026 Sep 8;17:1911635. doi: 10.3389/fneur.2026.1911635 (PMC13593471; doi:10.3389/fneur.2026.1911635)
Supplement: Supplementary file 1 [file Table_1.DOCX]

**Supplementary Table S1. Search strategy**

**Topic:** Robot-assisted rehabilitation for balance function and postural control after stroke: an umbrella review and evidence map of systematic reviews.

**Search date:** March 30, 2026. Searches were conducted from database inception to this date. No language restriction was applied at the search stage. Database-specific controlled vocabulary and free-text terms were adapted for each source.

## **1. PubMed**

| **Database** | **Search period** | **Records identified** |
| --- | --- | --- |
| PubMed | Inception to March 30, 2026 | 137 |

Search strategy:

#1 Stroke terms:
("Stroke"[Mesh] OR "Cerebrovascular Disorders"[Mesh] OR stroke[tiab] OR strokes[tiab] OR poststroke[tiab] OR post-stroke[tiab] OR "cerebrovascular accident"[tiab] OR "cerebrovascular accidents"[tiab] OR CVA[tiab] OR hemiplegia[tiab] OR hemiparesis[tiab])

#2 Robot-assisted rehabilitation terms:
("Robotics"[Mesh] OR "Robotic Surgical Procedures"[Mesh] OR robot*[tiab] OR robotic*[tiab] OR "robot-assisted"[tiab] OR "robot assisted"[tiab] OR "robot-assisted rehabilitation"[tiab] OR "robot-assisted therapy"[tiab] OR "robot-assisted gait training"[tiab] OR "robotic gait training"[tiab] OR "electromechanical-assisted"[tiab] OR "electromechanical assisted"[tiab] OR exoskeleton*[tiab] OR "wearable robot"[tiab] OR "wearable robots"[tiab] OR Lokomat[tiab] OR "Gait Trainer"[tiab] OR "G-EO"[tiab] OR end-effector[tiab] OR "lower limb rehabilitation robot"[tiab] OR "lower-limb rehabilitation robot"[tiab])

#3 Balance/postural-control terms:
("Postural Balance"[Mesh] OR balance[tiab] OR "postural control"[tiab] OR "postural stability"[tiab] OR "postural balance"[tiab] OR "Berg Balance Scale"[tiab] OR BBS[tiab] OR "Timed Up and Go"[tiab] OR TUG[tiab] OR TUGT[tiab] OR "Fugl-Meyer balance"[tiab] OR FMB[tiab] OR "Functional Reach Test"[tiab] OR FRT[tiab] OR "Postural Assessment Scale"[tiab] OR PASS[tiab] OR "center of pressure"[tiab] OR "centre of pressure"[tiab] OR COP[tiab] OR "postural sway"[tiab])

#4 Review terms:
("Systematic Review"[Publication Type] OR "Meta-Analysis"[Publication Type] OR "systematic review"[tiab] OR "meta-analysis"[tiab] OR metaanalysis[tiab] OR "network meta-analysis"[tiab] OR overview[tiab] OR umbrella[tiab])

Final search:
#1 AND #2 AND #3 AND #4
Records identified: 137

## **2. Embase**

| **Database** | **Search period** | **Records identified** |
| --- | --- | --- |
| Embase | Inception to March 30, 2026 | 102 |

Search strategy:

#1 Stroke terms:
'exp stroke'/exp OR 'cerebrovascular disease'/exp OR stroke:ti,ab,kw OR strokes:ti,ab,kw OR poststroke:ti,ab,kw OR 'post-stroke':ti,ab,kw OR 'cerebrovascular accident':ti,ab,kw OR 'cerebrovascular accidents':ti,ab,kw OR cva:ti,ab,kw OR hemiplegia:ti,ab,kw OR hemiparesis:ti,ab,kw

#2 Robot-assisted rehabilitation terms:
'robotics'/exp OR robot*:ti,ab,kw OR robotic*:ti,ab,kw OR 'robot assisted':ti,ab,kw OR 'robot-assisted':ti,ab,kw OR 'robot-assisted rehabilitation':ti,ab,kw OR 'robot-assisted therapy':ti,ab,kw OR 'robot-assisted gait training':ti,ab,kw OR 'robotic gait training':ti,ab,kw OR 'electromechanical assisted':ti,ab,kw OR 'electromechanical-assisted':ti,ab,kw OR exoskeleton*:ti,ab,kw OR 'wearable robot':ti,ab,kw OR 'wearable robots':ti,ab,kw OR lokomat:ti,ab,kw OR 'gait trainer':ti,ab,kw OR 'g-eo':ti,ab,kw OR 'end effector':ti,ab,kw OR 'end-effector':ti,ab,kw OR 'lower limb rehabilitation robot':ti,ab,kw OR 'lower-limb rehabilitation robot':ti,ab,kw

#3 Balance/postural-control terms:
'postural balance'/exp OR balance:ti,ab,kw OR 'postural control':ti,ab,kw OR 'postural stability':ti,ab,kw OR 'postural balance':ti,ab,kw OR 'berg balance scale':ti,ab,kw OR bbs:ti,ab,kw OR 'timed up and go':ti,ab,kw OR tug:ti,ab,kw OR tugt:ti,ab,kw OR 'fugl-meyer balance':ti,ab,kw OR fmb:ti,ab,kw OR 'functional reach test':ti,ab,kw OR frt:ti,ab,kw OR 'postural assessment scale':ti,ab,kw OR pass:ti,ab,kw OR 'center of pressure':ti,ab,kw OR 'centre of pressure':ti,ab,kw OR cop:ti,ab,kw OR 'postural sway':ti,ab,kw

#4 Review terms:
'systematic review'/exp OR 'meta analysis'/exp OR 'systematic review':ti,ab,kw OR 'meta-analysis':ti,ab,kw OR metaanalysis:ti,ab,kw OR 'network meta-analysis':ti,ab,kw OR overview:ti,ab,kw OR umbrella:ti,ab,kw

Final search:
#1 AND #2 AND #3 AND #4
Records identified: 102

## **3. Cochrane Library**

| **Database** | **Search period** | **Records identified** |
| --- | --- | --- |
| Cochrane Library | Inception to March 30, 2026 | 39 |

Search strategy:

#1 Stroke terms:
MeSH descriptor: [Stroke] explode all trees OR MeSH descriptor: [Cerebrovascular Disorders] explode all trees OR stroke:ti,ab,kw OR strokes:ti,ab,kw OR poststroke:ti,ab,kw OR "post-stroke":ti,ab,kw OR "cerebrovascular accident":ti,ab,kw OR hemiplegia:ti,ab,kw OR hemiparesis:ti,ab,kw

#2 Robot-assisted rehabilitation terms:
MeSH descriptor: [Robotics] explode all trees OR robot*:ti,ab,kw OR robotic*:ti,ab,kw OR "robot-assisted":ti,ab,kw OR "robot assisted":ti,ab,kw OR "robot-assisted rehabilitation":ti,ab,kw OR "robot-assisted therapy":ti,ab,kw OR "robot-assisted gait training":ti,ab,kw OR "robotic gait training":ti,ab,kw OR "electromechanical-assisted":ti,ab,kw OR "electromechanical assisted":ti,ab,kw OR exoskeleton*:ti,ab,kw OR Lokomat:ti,ab,kw OR "Gait Trainer":ti,ab,kw OR "G-EO":ti,ab,kw OR end-effector:ti,ab,kw OR "lower limb rehabilitation robot":ti,ab,kw

#3 Balance/postural-control terms:
MeSH descriptor: [Postural Balance] explode all trees OR balance:ti,ab,kw OR "postural control":ti,ab,kw OR "postural stability":ti,ab,kw OR "postural balance":ti,ab,kw OR "Berg Balance Scale":ti,ab,kw OR BBS:ti,ab,kw OR "Timed Up and Go":ti,ab,kw OR TUG:ti,ab,kw OR TUGT:ti,ab,kw OR "Fugl-Meyer balance":ti,ab,kw OR FMB:ti,ab,kw OR "Functional Reach Test":ti,ab,kw OR FRT:ti,ab,kw OR "Postural Assessment Scale":ti,ab,kw OR PASS:ti,ab,kw OR "center of pressure":ti,ab,kw OR "centre of pressure":ti,ab,kw OR COP:ti,ab,kw OR "postural sway":ti,ab,kw

#4 Review terms:
"systematic review":ti,ab,kw OR "meta-analysis":ti,ab,kw OR metaanalysis:ti,ab,kw OR "network meta-analysis":ti,ab,kw OR overview:ti,ab,kw OR umbrella:ti,ab,kw

Final search:
#1 AND #2 AND #3 AND #4
Records identified: 39

## **4. Web of Science**

| **Database** | **Search period** | **Records identified** |
| --- | --- | --- |
| Web of Science | Inception to March 30, 2026 | 112 |

Search strategy:

#1 Stroke terms:
TS=(stroke OR strokes OR poststroke OR "post-stroke" OR "cerebrovascular accident" OR "cerebrovascular accidents" OR CVA OR hemiplegia OR hemiparesis)

#2 Robot-assisted rehabilitation terms:
TS=(robot* OR robotic* OR "robot-assisted" OR "robot assisted" OR "robot-assisted rehabilitation" OR "robot-assisted therapy" OR "robot-assisted gait training" OR "robotic gait training" OR "electromechanical-assisted" OR "electromechanical assisted" OR exoskeleton* OR "wearable robot" OR "wearable robots" OR Lokomat OR "Gait Trainer" OR "G-EO" OR "end-effector" OR "end effector" OR "lower limb rehabilitation robot" OR "lower-limb rehabilitation robot")

#3 Balance/postural-control terms:
TS=(balance OR "postural control" OR "postural stability" OR "postural balance" OR "Berg Balance Scale" OR BBS OR "Timed Up and Go" OR TUG OR TUGT OR "Fugl-Meyer balance" OR FMB OR "Functional Reach Test" OR FRT OR "Postural Assessment Scale" OR PASS OR "center of pressure" OR "centre of pressure" OR COP OR "postural sway")

#4 Review terms:
TS=("systematic review" OR "meta-analysis" OR metaanalysis OR "network meta-analysis" OR overview OR umbrella)

Final search:
#1 AND #2 AND #3 AND #4
Records identified: 112

**Abbreviations:** BBS, Berg Balance Scale; CCA, corrected covered area; COP, center of pressure; FMB, Fugl-Meyer balance; FRT, Functional Reach Test; PRISMA, Preferred Reporting Items for Systematic Reviews and Meta-Analyses; RAGT, robot-assisted gait training; SFRT, Standing Functional Reach Test; TUG/TUGT, Timed Up and Go/Timed Up and Go Test.

**Note:** The search syntax was adapted according to each database interface. The record numbers correspond to the final searches used for the PRISMA flow diagram.
